# The role of apelin in cancer

**DOI:** 10.3389/fonc.2025.1683865

**Published:** 2025-10-03

**Authors:** Yuki Agarwala, Rohini Sharma

**Affiliations:** ^1^ Imperial College School of Medicine, London, United Kingdom; ^2^ Department of Surgery and Cancer, Imperial College, London, United Kingdom

**Keywords:** cancer, biomarkers, cell proliferation, angiogenesis, apelin

## Abstract

Apelin is widely expressed in the body. Apelin is secreted as a propeptide and is proteolytically cleaved into six main isoforms. As an adipokine, apelin has a cardio- and neuroprotective role and is involved in angiogenesis in healthy tissue. Recently, numerous roles of endothelial cell-derived apelin on cancer cells have been uncovered, including angiogenesis, cell proliferation, and cellular invasion. Studies have reported elevated apelin levels in tumor tissue of patients with cancer compared to healthy controls, highlighting its role in determining prognosis. Research suggests that inhibiting apelin reduces tumor invasiveness and angiogenesis and further mechanistic studies are required to realize the full potential of therapeutics targeting apelin in malignancy.

## Introduction

Apelin (APLN) is an adipokine that is ubiquitously expressed in healthy tissues and plays an integral role in homeostasis through regulating angiogenesis, cellular metabolism, and proliferation, among many others (1–3). These functions of APLN are critical to its contribution to tumorigenesis, but recent studies have revealed contradictory results in regard to the effect of APLN on recruiting immune cells into the tumor microenvironment in murine models of different cancers (4, 5). Interestingly, such contrary effects were also seen for migration and metastasis, where APLN either promoted or reduced tumor growth depending on the cancer type (6, 7). Because of the role of APLN in cancer, tissue and serum APLN have both been studied as prognostic biomarkers, and the protein shows promise as a potential anti-tumorigenic agent (3). Several studies have shown reduced tumor growth when compounds targeting APLN are used in conjunction with other therapeutic agents (7). While the effect of APLN varies significantly based on the cancer type, studies highlight the potential application of APLN in the prognostic and therapeutic settings, which is the subject of this narrative.

## Structure of apelin

**Figure 1:**
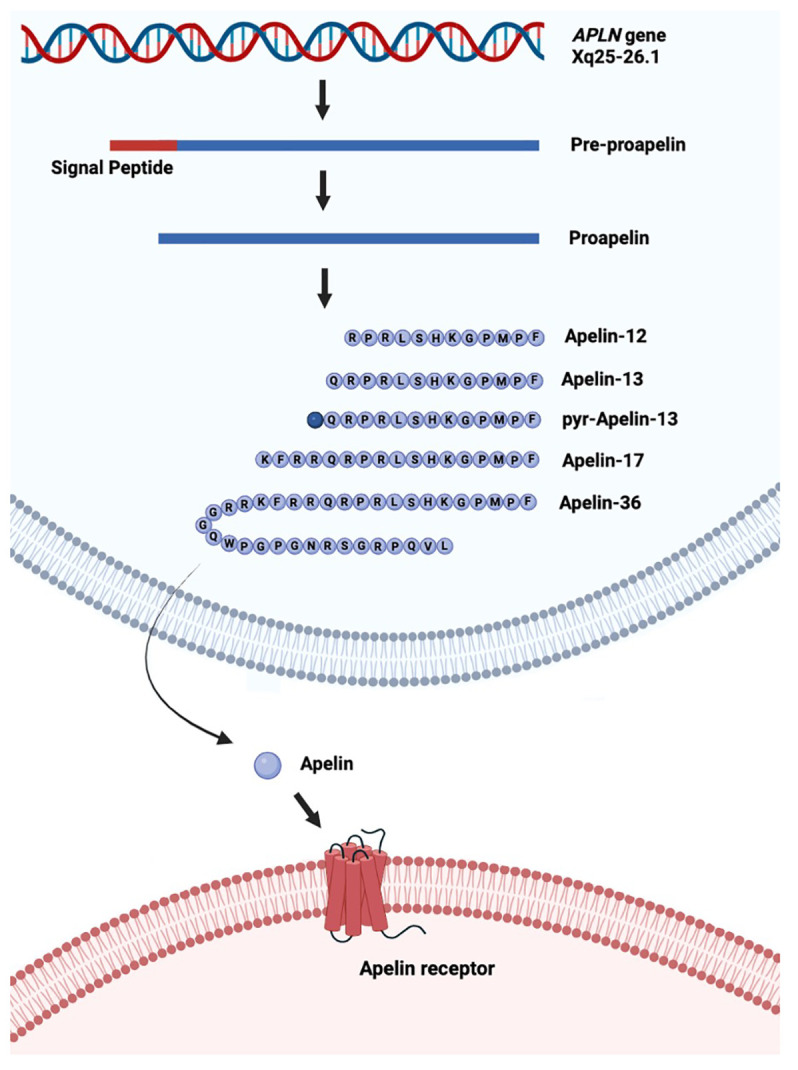
Schematic of production of the different isoforms of apelin from the *APLN* gene. The *APLN* gene is transcribed into pre-proapelin from which the signal peptide is cleaved to produce proapelin. Proapelin is subsequently modified into one of five different isoforms of apelin (APLN), which leave the cell and bind to the apelin receptor (APLNR). Pyr: pyroglutamated. Created in Biorender.com and based on Melgar-Lesmes et al. (12) and Murali and Aradhyam (13) under CC BY 4.0.

The *APLN* gene is located on chromosome Xq25-26.1 and contains three exons, which result in two transcriptional products (8). *APLN* encodes APLN, an adipokine, or cytokine produced by adipose tissue, and is involved in various processes including energy metabolism and angiogenesis (9). Apelin is secreted as a propeptide of 77 amino acids, which is proteolytically cleaved, resulting in biologically active C-terminal peptides (10). The main isoforms of apelin include APLN-36, APLN-17, APLN-13, pyroglutamated-13, and pyroglutamated-12 (11) (**Figure 1**). The amino acid sequence is evolutionarily conserved, with APLN-17 and APLN-13 being 100% homologous between human, rat, mouse, and bovine (14). APLN is the ligand for the apelin receptor (APLNR), a seven-transmembrane G protein-coupled receptor. APLNR is encoded on chromosome 11q12, and its amino acid sequence is highly conserved between species. It shares its sequence with the angiotensin type-1 receptor but is not activated by angiotensin II (8).

## Function of apelin in healthy tissue

APLN/APLNR mRNA is widely expressed, with the highest expression observed in cardiac tissue, lung, stomach, and central and peripheral nervous systems, among other areas (3). APLN has a physiological role in homeostasis including cellular migration and proliferation, angiogenesis, inflammation, and metabolism (1, 2). The APLN/APLNR system is critical to the regulation of blood pressure as APLN opposes the effect of the angiotensin II–renin system (15). Receptors of apelin and angiotensin are co-expressed and may have regulatory effects on each other (16). As an adipocytokine, APLN is released by adipose tissue and is implicated in metabolism. Plasma concentrations are increased in obese patients and patients with type II diabetes mellitus (17). APLN reduces plasma glucose concentration through insulin-dependent and -independent pathways endogenously (18), but the effect of administering APLN on diabetic control externally remains unknown.

Endothelial cells also express APLN, which plays a critical role in angiogenesis. In an *in vitro* model of human umbilical vein endothelial cells, the addition of vascular endothelial growth factor (VEGF) upregulated APLN and induced both cellular proliferation and increased blood vessel diameter (19). In addition to angiogenesis, APLN also increased cell-to-cell contact, inducing the assembly of endothelial cells (19). ML221, an APLNR antagonist, reduces the formation of neovascular tufts (20), indicating the importance of APLN homeostasis for the regulation of angiogenesis in healthy tissue. APLN has also been found to have neuroprotective effects, playing an integral function in motor neuron survival. In cultured primary rodent cortical neurons, APLN activates both the PI3K/Akt and Raf/ERK cascades to protect against N-methyl-D-asparate excitotoxicity. Similar effects were demonstrated *in vivo*, with APLNR knock-out (KO) mice exhibiting increased disease progression in amyotrophic lateral sclerosis compared to APLNR-expressing mice (21). These studies show the extensive effects APLN has on healthy tissue, and these pathways are also crucial for the role of APLN in cancer.

## Role of apelin in cancer

In malignancy, APLN has been implicated in cancer growth, mortality, and prognosis through a wide range of mechanisms. This is predominantly due to the effect of APLN on the tumor microenvironment through angiogenesis, migration, and invasion (22).

### Effect of APLN on angiogenesis and proliferation is dependent on the tumor microenvironment

**Figure 2:**
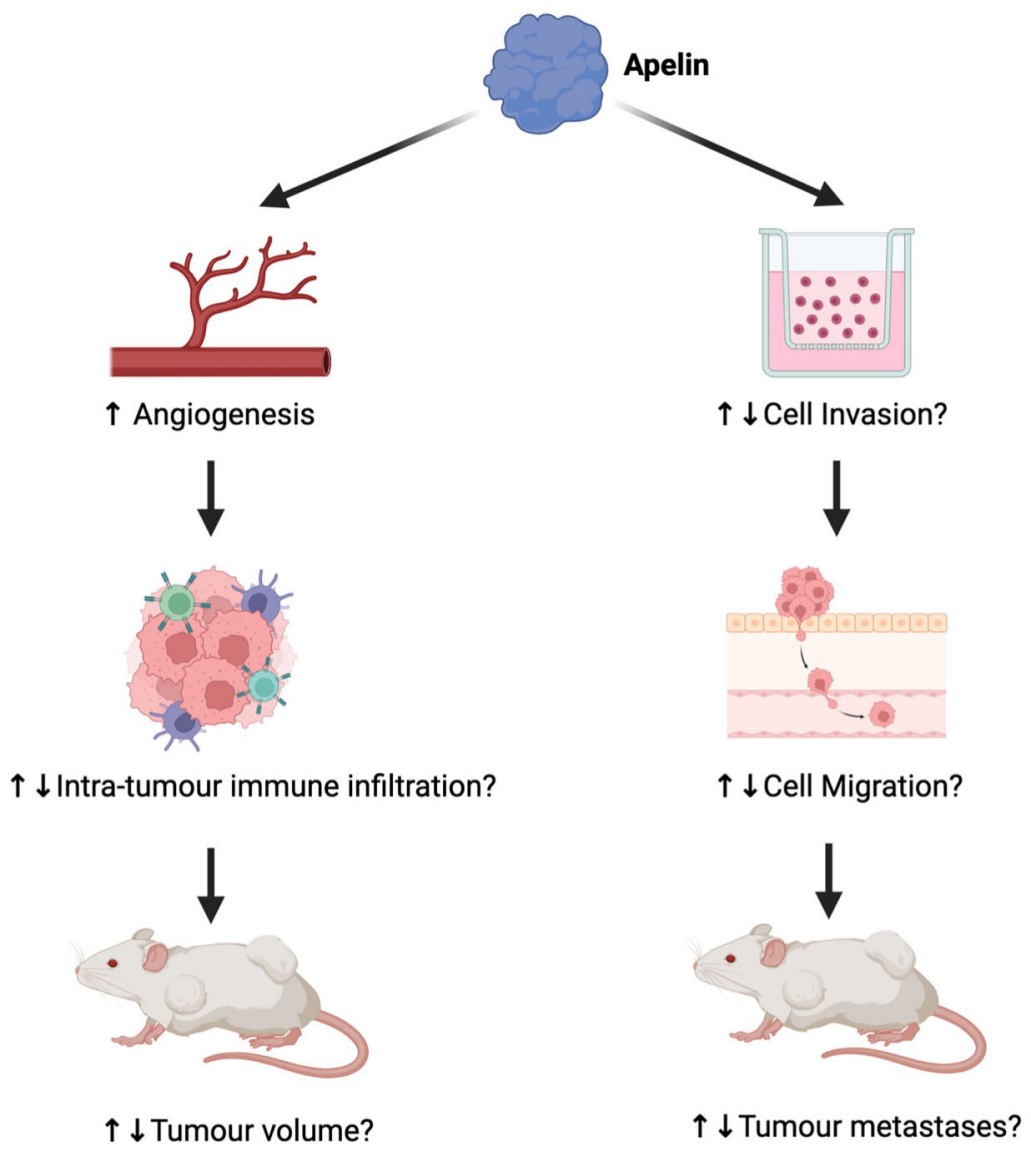
The differential impact of apelin (APLN) on tumors. While APLN increases angiogenesis, in some tumors, the effect has been considered part of functional maturation, causing reduced tumor growth (24), whereas in others, it has contributed to tumor growth (23). As a result, the impact of angiogenesis on intra-tumor immune infiltration and tumor volume varies greatly by tumor. The effect of APLN on cell invasion, migration, and therefore metastases also differs by tumor (6, 7, 25, 26). Figure created in Biorender.com.

APLN is integral to tumor development through its role in maintaining angiogenesis and tumor growth. For example, gene expression analysis illustrates significantly elevated APLN levels in non-small cell lung cancer compared to healthy lung tissue (23). However, the effect of APLN on tumor growth differs according to the immune infiltration within the specific tumor microenvironment (see **Figure 2**). To investigate the effect of APLN on tumor growth, various cancer cell lines were injected into wild-type (WT) and APLN KO mice. In a model of MC38 colon adenocarcinoma, a greater proportion of CD8^+^ and CD4^+^ T cells were distributed in the center of tumors of WT mice compared to APLN KO mice. CCL8 is a cytokine important for the recruitment of immune cells, and APLN was shown to induce CCL8 expression *in vitro*. Based on this, it was hypothesized that APLN enhances intratumoral immune infiltration in a CCL8-dependent manner in the MC38 cancer model (4). However, in a mouse model of glioblastoma (GBM), APLN KO mice had slower tumor growth than WT mice, which contradicts the results for the MC38 cancer model (5). Moreover, mice models of *Apln-*depleted E0771 mammary carcinoma had significantly increased intra-tumoral NK T cells, and decreased infiltration of polymorphonuclear myeloid-derived suppressor cells compared to WT mice (2). This discrepancy strongly suggests that the effect of APLN is dependent on the tumor microenvironment. Thus, further mechanistic research is required to determine the effect of APLN in different tumor microenvironments.

APLN has also been implicated in cell invasion and migration. When GBM14, a GBM xenograft cell line, was implanted into APLN KO mice, the invasive tumor volume significantly increased compared to APLN WT mice (7), suggesting a role in reducing cell invasion. In contrast, APLN supplementation stimulated invasion in colon cancer cell models (6) and increased lymphatic vessel densities in melanoma (25), underscoring the differential effects of APLN based on the tumor microenvironment (see **Figure 2**).

When four different colon cancer models of varying metastatic potential were stimulated with APLN *in vitro*, invasion increased in all cell lines. APLN increased the ratio of filamentous to monomeric actin, forming migratory protrusions, and also elevated levels of metalloproteinases, which are crucial for the degradation of the extracellular matrix (6). In OVCAR-4 and OCAR-8 high-grade serous ovarian cancer cells, APLN supplementation increased migration and cell invasion compared to control, and this effect was reversed with ML221. The metastatic properties are due to the activation of downstream STAT3. Compared to controls, mice injected with OVCAR-3 cells overexpressing *Apln* had higher numbers of large tumors on internal organs, indicating the role of APLN on cancer metastasis (26). While there is strong evidence of APLN regulating metastasis through matrix metalloproteinases (MMPs) and actin, the specific properties of certain tumors that may dictate the effects of APLN remain unknown.

## Apelin as a biomarker in cancer

### Tumor APLN as a prognostic marker

Elevated *APLN* and *APLNR* expression have been reported in many cancers including ovarian, prostate, liver, gastric, and lung cancers compared to healthy tissues (3). In a study of 124 patients with high-grade serous ovarian cancer, high tumor APLN expression was associated with a 14.7-month reduction in median overall survival compared to patients with low APLN expression (26). In another study of 60 patients with invasive breast carcinoma, patients with metastatic disease had significantly greater APLN expression in lymph nodes compared to patients without metastasis, suggesting the potential role of APLN as a driver of metastasis (27). High tumor APLN expression, not APLNR, was correlated with reduced overall survival and relapse-free survival in 518 patients with low-grade glioma (3) In contrast, in another study of 163 patients with GBM, neither *APLN* nor *APLNR* gene expression was correlated with overall survival or disease-free interval (3), suggesting inter-tumor variation. Therefore, the intra- and inter-tumor heterogeneity of APLN should be characterized if it were to be utilized clinically as a prognostic indicator of cancer.

### Serum APLN as a prognostic marker

Several studies have also investigated serum APLN as a non-invasive marker of prognosis. High APLN expression in serum has been correlated with shorter overall survival in renal cell carcinoma (28), gastric cancer (22), and muscle-invasive bladder cancer (29). However, elevated APLN in serum may not always be due to cancer. For example, in gastroesophageal cancer, high serum APLN was correlated with high C-reactive protein, suggesting that the elevated serum APLN could be due to systemic inflammation rather than cancer (30). Moreover, in patients with gastric cancer, both tumor and serum APLN expression were significantly elevated, but in patients with chronic gastritis, APLN expression in serum was also elevated. Together, these studies suggest that tumor APLN expression may be more accurate than serum measurements for prognostic purposes (22).

### APLN as a predictive marker

APLN has also been studied as a predictive indicator of treatment response. APLN is an adipokine thought to be increased in cases of obesity as insulin drives APLN expression. In a retrospective exploratory study on 62 patients with breast cancer, APLN expression and obesity were both found to be independently associated with increased aggressiveness of breast cancer and poor response to neoadjuvant chemotherapy (31). Because of the presence of few patients with diabetes in the patient cohort, the study had limited generalizability, but the interaction of obesity and APLN expression and its role in cancer therapies warrant further investigation. In a cohort of 13 patients with colorectal cancer given bevacizumab, non-responders were associated with significantly higher baseline *APLN* mRNA expression in the cancerous tissue, with poor progression-free survival compared to patients with low baseline *APLN* expression (32). This suggests that APLN can also be an indicator of treatment response, but further mechanistic research is required before clinical application.

## Apelin as an anti-tumorigenic therapeutic target

Considering the role of APLN in driving cell proliferation and angiogenesis, Hall et al. (33) demonstrated a dose-dependent response to APLN in the growth of cholangiocarcinoma. Treatment with ML221 using mouse models of cholangiocarcinoma demonstrated decreased tumor volume, decreased gene expression of proliferation (Ki-67), VEGF, and MMP compared to the untreated control (33). ML221 also inhibited migration and invasion in high-grade serous ovarian cancer (26), and MM54, another APJ antagonist, reduced the tumorigenic effects of APLN on melanoma (34).

Currently used GBM therapies such as the anti-VEGF antibody bevacizumab have been shown to decrease APLN mRNA expression and increase tumor volume in GBM murine models. Therefore, it was hypothesized that VEGF-A treatment resistance may be due to increased cell invasiveness from APLN reduction (7). Mice with orthotopic *p53* ^KO^ platelet-derived growth factor receptor-B GBM were synergistically given bevacizumab and APLN-F13A, an APLNR antagonist with partial agonist properties. Results showed decreased invasive volume and vascular density, and prolonged survival in the combination group compared to monotherapy (7). Similar results were seen in GL261 glioma murine models (5). APLN-F13A is a poorly understood compound, and the effects in the study may have been observed due to the partial agonistic properties of APLN-F13A, indicating that, in GBM, a complete antagonist of APLNR may be detrimental.

The studies show that the effect of APLNR modulation is different based on the tumor microenvironment, an important area for future research. Furthermore, the tumor heterogeneity of APLN and APLNR, as well as the percentage of patients who express increased APLN should be investigated as therapeutic efficacy targeting APLN will heavily depend on its expression. APLN affects multiple cancer pathways, and while initial data on its therapeutic efficacy alone or in combination are promising, further mechanistic studies are required to determine its efficacy.

## Conclusions

APLN was originally known for its role in angiogenesis in healthy tissues but has been found to significantly affect immune infiltration, epithelial–mesenchymal transition, tumor invasiveness, and a variety of other pathways in different cancers. Studies have shown that APLN can affect cancers differently according to the nature of their tumor microenvironment. Understanding their underlying mechanisms will be crucial to the application of APLN as a prognostic and predictive marker for different cancers. Mechanistic insight is also integral for shedding light on the potential for APLNR modulation for anti-cancer therapy.
